# New Pathways Identify Novel Drug Targets for the Prevention and Treatment of Alzheimer’s Disease

**DOI:** 10.3390/ijms24065383

**Published:** 2023-03-11

**Authors:** Botond Penke, Mária Szűcs, Ferenc Bogár

**Affiliations:** 1 Department of Medical Chemistry, University of Szeged, Dóm Square 8, H-6720 Szeged, Hungary; 2 ELKH-SZTE Biomimetic Systems Research Group, Eötvös Loránd Research Network (ELKH), Dóm Square 8, H-6720 Szeged, Hungary

**Keywords:** Alzheimer’s disease, toxic amyloids, Aβ, tau, genetics, amyloid clearance, vascular dysfunction, neuroinflammation, heat shock proteins, drug targets

## Abstract

Alzheimer’s disease (AD) is an incurable, progressive neurodegenerative disorder. AD is a complex and multifactorial disease that is responsible for 60–80% of dementia cases. Aging, genetic factors, and epigenetic changes are the main risk factors for AD. Two aggregation-prone proteins play a decisive role in AD pathogenesis: β-amyloid (Aβ) and hyperphosphorylated tau (pTau). Both of them form deposits and diffusible toxic aggregates in the brain. These proteins are the biomarkers of AD. Different hypotheses have tried to explain AD pathogenesis and served as platforms for AD drug research. Experiments demonstrated that both Aβ and pTau might start neurodegenerative processes and are necessary for cognitive decline. The two pathologies act in synergy. Inhibition of the formation of toxic Aβ and pTau aggregates has been an old drug target. Recently, successful Aβ clearance by monoclonal antibodies has raised new hopes for AD treatments if the disease is detected at early stages. More recently, novel targets, e.g., improvements in amyloid clearance from the brain, application of small heat shock proteins (Hsps), modulation of chronic neuroinflammation by different receptor ligands, modulation of microglial phagocytosis, and increase in myelination have been revealed in AD research.

## 1. Introduction

Neurodegenerative diseases (NDDs) represent a big part of neurological disorders. NDDs are characterized by the loss of synapses and neurons in the central nervous system (CNS). Neuronal loss often generates the decline of cognitive functions and dementia. Many NDDs have a common central neuropathological event: misfolded, toxic protein aggregates (amyloids) are accumulated in the CNS. NDDs can be regarded as protein homeostasis disorders: the level of several aggregation-prone proteins is increased, and subsequent small conformational changes result in the accumulation of pathogenic, β-structured amyloid proteins. Many amyloid protein structures are self-replicating, display prionoid character, and their aggregated form is propagated from cell to cell (transmissible pathology) [1]. The prionoid propagation of several amyloids (e.g., tau protein and α-synuclein) has been proven. The precise molecular mechanism of the conversion of a nontransmissible protein to the pathogenic prionoid form is not completely understood. Although the native, functionally-folded proteins possess important physiological functions, their misfolded amyloid aggregates are toxic to brain cells. The amyloid structure fundamentally differs from the globular state of these proteins and has remarkable similarities in the molecular and supramolecular organization [2]. Medical research has revealed common disease pathways among NDDs [3]; the most important of them is the accumulation of toxic, misfolded proteins.

Distinct NDDs are coupled to the accumulation of different misfolded amyloid proteins: AD to β-amyloid (Aβ), hyperphosphorylated tau (pTau) and other proteins, Parkinson’s disease (PD) to α-synuclein (α-syn), Huntington’s disease (HD) to huntingtin, amyotrophic lateral sclerosis (ALS) to SOD-1 or TDP-43, and prion diseases to prion proteins. Amyloid accumulation may also occur in the parenchymal organs of the periphery (e.g., heart and kidneys) and cause the progress of serious diseases. Existing treatments for NDDs are limited and mainly address symptoms rather than causes of the disease. Very recent studies have shown that these diseases are complex and multifactorial, presenting hurdles for discovering novel therapies. In the recent genomic era, novel experimental results have opened up the opportunity for using genetic, epigenetic, transcriptomic, proteomic, metabolomics, and lipidomic data for designing novel drugs for the treatment of NDDs. Developing preventive and, ultimately, disease-modifying therapies for slowing the progression of neurodegeneration in AD and other NDDs seems to be one of the greatest medical needs of our time.

The present review first summarizes our most important knowledge of AD (pathology, genetic background, different hypotheses of the progression of AD, and novel methods for early diagnosis). In the second part, we show novel targets for preventing and/or slowing the progress of AD (e.g., increase in amyloid clearance, inhibition of amyloid formation and accumulation, and modulation of neuroinflammation).

## 2. Alzheimer’s Disease

### 2.1. Pathology and Classification of AD. Aging and Dementia. The Main Risk Factors of AD

Presently, AD is an incurable and progressive neurodegenerative disorder characterized by mixed proteinopathy, progressive dysfunction and loss of synapses, behavioral dysfunction, memory loss, and rapid cognitive decline [4]. Clinicopathologically, AD is a heterogeneous, multifactorial disease with different pathobiological subtypes. AD is rather a spectrum, a continuum from the preclinical, asymptotic phase via mild cognitive impairment to severe AD dementia [4]. Extremely complex, interrelated, and destructive processes lead to cell death and dementia. Novel strategies emphasize the importance of multitarget therapies for AD treatment due to the heterogeneity of the disease [5].

Excellent reviews based on the results of systematic postmortem analyses summarize the pathology of AD [5,6,7,8,9]. The two most important pathological hallmarks of AD brains were described by A. Alzheimer in 1907. These are the formation of extracellular Aβ deposits (plaques) and intracellular neurofibrillary tangles (NFTs). Different Aβ deposits have been found in the brain and cognitively normal individuals. The distribution of amyloid plaques represents the major difference between cognitively normal individuals and AD patients. All types of cerebral, nonvascular Aβ deposits are referred to as “senile plaques.” Cerebral Aβ deposition shows different phases [10]. Diffuse plaques occur in brain regions in β-amyloidosis at early stages, and cored neuritic plaques only occur in later stages of Aβ deposition. Diffuse plaques appear in all phases of deposition. The presence of diffuse plaques is not coupled to AD, while neuritic plaques have been shown to be associated with dementia. Aβ deposits mainly have a complex structure that contains several coaggregating proteins (Apolipoprotein E: APOE, Cathepsin D, and Clusterin), as well as dystrophic neurites and microglia. Other morphological hallmarks are vascular amyloid deposits, neuroinflammation, neuronal loss, and astrogliosis. 

AD is classified into early onset (EOAD) and late-onset (LOAD) forms. Both forms possess common pathological features. The symptoms appear before the age of 65 in EOAD and after 65 in LOAD. The different clinical forms of AD have been classified into four subtypes based on the distribution of tau pathology and neuronal loss: typical, minimal atrophy, limbic predominant, and hippocampal spearing subtypes. Deposition of Aβ to extracellular plaques follows different pathways and shows distinct patterns. The specific pattern of tau pathology correlates better than that of Aβ deposition with the clinical symptoms of AD patients (cognitive impairment and memory loss). AD starts when both amyloid and tau pathologies overlap [11]. Several other heterogeneous AD variants have recently been identified based on the atrophy of different neuronal networks. Age-related copathologies are also frequent (e.g., Lewy bodies and hippocampal sclerosis), which makes it difficult to understand the pathomechanism of AD in individual cases [9].

The neuropathological staging of AD was already performed in 1991 [12] based on the development of tau (NFT) pathology in the brain (stages I to VI). Braak stages show some correlation to the clinically observed severity of dementia. Thal and coworkers demonstrated that the spreading of amyloid deposits in the brain could be predicted and occurs in five stages [13]. Aβ plaques appear in the neocortex in phase 1 (cognitive functions, working memory, speech perception, and language skill). Later, Aβ deposition also occurs in allocortical brain regions (phase 2). Subcortical areas (striatum and basal cholinergic forebrain nuclei) are involved in further depositions (phase 3). In phase 4, several brainstem nuclei (vital functions and relaying neuronal impulses) are affected by the deposition of a high density of plaques, and their level is in good correlation with the symptoms of dementia. Finally, phase 5 is characterized by amyloid depositions in the cerebellum (movement coordination). Biomarkers can monitor the deposition of Aβ plaques and tau in living patients. These studies demonstrate that Aβ and tau pathologies already start decades before symptoms of cognitive decline in AD patients (biomarker-based diagnosis of the preclinical stage, see Section 2.5).

AD is the most common cause of dementia. Although the basic pathophysiological mechanism of AD is not yet clear, many indications address the importance of aging, as well as genetic and environmental factors. Aging seems to be the most important risk factor of AD (“the neurobiology of aging and AD is walking down the same road”) [6]. Some of the hallmarks of aging (e.g., mitochondrial dysfunction, low-grade chronic inflammation, and loss of proteostasis) overlap with the hallmarks of AD. Aging, genetic background, and epigenetic changes are the main risk factors for AD. 

Normal aging of the brain induces many changes. There are gross and microscopic alterations in the brain structure and metabolism (e.g., volume loss, demyelination, enlargement of ventriculi, dysfunction of the cholinergic system, decreased ligand binding affinity of several receptors, alteration in gene expression, a decrease in synaptic function, lipofuscin accumulation, disturbances of the blood-brain-barrier (BBB) function, an increase in cellular waste, etc.) [14]. Tissue atrophy, alteration in certain neurotransmitters, and dyshomeostasis of the cellular environment accompany normal aging and ultimately result in cognitive decline [14].

Dementia, a common sign of NDDs, is not a part of normal aging. It is very different from the cognitive decline that manifests during normal aging. Dementia is a syndrome of acquired, progressive cognitive impairment, frequently accompanied by depression, loss of memory, orientation, etc., which symptoms result in disruption of basic self-care. There are several modifiable risk factors for dementia, e.g., smoking, diabetes, obesity, depression, and physical inactivity [15]. 

Aging is accompanied by more and more frequent misfolding and aggregation of proteins [16,17]. Active balancing of protein synthesis and degradation are critical processes in the cells [18]. An adaptive network of functions called protein quality control (PQC) has evolved over millions of years to maintain cellular protein homeostasis (proteostasis). Different chaperones (e.g., heat shock proteins, Hsps) control the correct folding and aggregation of proteins. The vulnerability of the aging brain tissue may be demonstrated by the presence of suboptimal levels of proteostasis components (low levels of aggregation protectors and high levels of aggregation promoters) [19]. If PQC fails, a misfolding process results in the formation of pathogenic aggregates that are divided into two groups: unordered amorphous and rather ordered fibrillar amyloids [16]. Several disease-related amyloids (e.g., β-amyloid peptides) possess a high propensity for irreversible aggregation. It has been uncovered that age-dependent protein aggregation is a common feature of aging [17]. Structural alteration of the peptidic backbone might be one of the reasons for protein aggregation [20]. Spontaneous isomerization and epimerization of the aspartyl residue to D-Asp and L- and D-isoAsp results in modified protein structures with high protease resistance [21,22]. Hundreds of proteins become highly insoluble during the aging process [23]. Results of systematic postmortem analysis of AD brains demonstrated that two amyloid proteins play a decisive role in the development of AD: Aβ (mainly the 1–40 and 1–42 amino acid peptides) and pTau (see Section 2.3).

### 2.2. Genetic Background of AD, the Multiplex Model

Both forms of AD show high heritability [24]. The estimated heritability is over 90% for EOAD, and it is in the range of 60–80% for LOAD. Mutations in the amino acid sequence of the amyloid precursor protein (APP) and presenilin (PS) genes (presenilin 1 and 2 proteins are important for APP processing to Aβ) are causative factors for EOAD [8]. About 60 highly penetrant APP mutations have been discovered that are involved in the progress of EOAD. (Interestingly, a protective mutation of APP that decreases Aβ aggregation was found in the Iceland population [25]). PSEN mutations are responsible for 80% of EOAD cases. Over 350 mutations have been identified so far. The amyloid hypothesis is based on the strong genetic evidence of EOAD and Down syndrome. Altered APP processing and Aβ overproduction are in the background of AD.

The genetics of LOAD is much more complex than that of EOAD, and it is the result of combined influences of multiple genetic loci or polygenic effects. Genetic studies have demonstrated that AD is a multicomponent disease [26]. Until 2020, over 50 genetic risk factors have been identified that are responsible for LOAD (multiplex model of AD) [26]. APOE4 was proven to be the strongest single risk factor for LOAD (cholesterol synthesis and transport). It has recently been demonstrated that ApoE4 significantly increases tau pathogenesis and tau-associated neurodegeneration. Global ApoE deficiency is strongly protective [27]. Very recently, it was found that deletion of neuronal ApoE4 drastically reduces tau pathology in pTau overproducing PS19-ED mice [28]. These studies provide evidence that ApoE4 influences a multitude of events in AD progression (Aβ and tau accumulation, neuronal hyperexcitability, and myelin deficits). It was also reported that ApoE4 does not directly drive neurodegeneration, but microglia may mediate the effect of ApoE4 [29]. Interestingly, the majority of the genes/loci are associated with immune functions. For example, the TREM2 (triggering receptor expressed on myeloid cells 2) gene was identified to play a key role in microglia and macrophage function [30]. Genome-wide association studies (GWAS) enable the study of tens of thousands of patients and millions of genetic variations [31]. A two-stage GWAS was performed with 111,326 clinically diagnosed AD patients and 677,663 control individuals [32]. In these studies, 75 AD risk loci were found, of which 42 were new. Over 130 AD-associated loci were identified by GWAS, among them APOE4, TREM2, CR1, CD33, CLU, BIN1, CD2AP, PICALM, SORL1, SP11, RIN3, and more genes in another study [33]. Pathway analysis methods were used for studying genomic associations for identifying disease-relevant processes [33]. It was found that the majority of the genes are associated with immune functions. According to the multiplex model, besides immunity and inflammation, other pathways (for example, endocytosis, cholesterol metabolism and transport, Aβ clearance, tau processing, autophagy, and vascular factors) also participate in AD initiation. Genetic approaches have provided the first convincing evidence that the immune system plays a decisive role in the progression of AD.

Beyond inherited genetics, somatic mutations and diverse epigenetic mechanisms (DNA methylation, histone modification, chromatin remodeling, and long, noncoding RNAs) may participate in aging and neurodegeneration of the brain [34].

### 2.3. Amyloid Structures. Physiological and Pathophysiological Role of the Aβ and Tau Proteins

Amyloidogenic processing of APP provides a heterogeneous mixture of Aβ peptides of 37 to 43 amino acids. The physiological (neuroprotective) role of the monomeric Aβ 1–42 peptide has been widely reviewed [35,36,37]. Very recently, a Special Issue of Frontiers in Molecular Neuroscience has been published dealing with the physiological and pathological role of Aβ in detail [38]. 

Monomeric Aβ 1–42 is a neuropeptide, a physiological neuroprotector. Aβ 1–42 regulates synaptic function, neural circuitry, organelle trafficking, neurogenesis, neuroinflammation, and cognitive processes in picomolar concentrations [38]. Aβ 1–42 in low concentration suppresses microbial infections and can seal leaks in the blood-brain barrier, BBB (“vascular plug”) [37,39]. In physiological concentrations, Aβ maintains angiogenesis and vascularization, protects the BBB, promotes recovery after brain injury, and acts as a tumor suppressor [39]. Aβ can be produced intracellularly by APP cleavage. External Aβ can enter the cells by receptor-mediated internalization [40]. Aβ monomers start to aggregate into different Aβ oligomers over a critical concentration [41]. Intracellular Aβ also participates in neurodegenerative processes [42]. The aggregation process provides different Aβ assemblies; in the first step, oligomers (oAβ):

native Aβ monomer → partially folded monomer → transient oAβ → protofibrils (β-sheet → structured oAβ → fibrils (cross-β) → big aggregates, plaques.

Amyloid aggregation has been experimentally studied under in vivo conditions [43]. Amyloid proteins interact with other proteins (cross-seeding) [44]. It was found that cross-seeding plays a key role in amyloid formation. Lipids (e.g., gangliosides and cholesterol) and divalent metal ions (e.g., zinc, copper, and iron) are good nucleators for Aβ (Section 2.4) [45]. Experiments (TEM studies of Aβ aggregates) demonstrated that several D-amino acids (D-Ala, D-Phe, D-Glu, and D-Asp) and DL-selenoMet initiate Aβ aggregation (enantiomeric-induced aggregation [46]). Most of the L-amino acids did not affect amyloid aggregation. Selenium nanoparticles inhibited enantiomeric-induced amyloid aggregation and could be beneficial compounds for AD treatment.

It was demonstrated that protein insolubility and aggregation might be critical for mediating the pathogenesis of NDDs in older ages. Thus, inhibition of the aggregation process seems to be a good target of drug design against NDDs. The very recent development of techniques, such as cryo-EM (cryo-electron microscopy), solid-state NMR (solid-state nuclear magnetic resonance), and AFM (atomic force microscopy), has opened up new ways of understanding amyloid structure [2,47]. Cryo-EM data can be integrated into comparative morphometric AFM image analysis of amyloid fibrils [48]. Cryo-EM studies demonstrated the complex structure and peptide conformation of Aβ fibrils isolated from the brain of AD patients [49,50,51]. 

Animal studies demonstrate that oAβ is sufficient and necessary for AD-associated neurodegeneration [52,53]. Aβ oligomers have toxic effects on the brain [41]). They were shown to inhibit axonal transport, cause synaptic damage and dysfunction of neuronal plasticity, Ca^2+^-dyshomeostasis, oxidative stress, ER stress (endoplasmic reticulum stress), and selective neuronal death. One of the most important effects of oAβ is the promotion of tau-hyperphosphorylation. Oligomeric Aβ interacts with the lipid membrane components (e.g., ganglioside GM1) and directly binds to different receptors [54]. Not all the binding proteins are genuine receptors since Aβ has an intrinsically disordered structure and can associate with many proteins. 

The most important pathogenic events induced by toxic oAβ are (1) stimulation of tau-hyperphosphorylation, (2) impairment of mitochondrial function, (3) disruption of Ca^2+^ and protein homeostasis, and (4) induction of autophagy dysfunction. [40]. 

Another key player in AD progression is the tau protein. Alternative splicing of the human microtubule-associated protein tau (MAPT) gene provides six tau isoforms, which may aggregate into oligomers and filaments [41]. Tau shows a large number of post-translational modifications [55].

The physiological role of tau proteins is widely reviewed [56,57]. As a multifunctional protein, tau plays an important role in physiological processes [41,58]. Tau participates in maintaining DNA integrity, protects and regulates microtubules and axonal transport, interacts with cytoskeletal proteins (e.g., actin and spectrin), and regulates the shape of the cells [39]. Native tau is necessary for normal myelination and also regulates transcription at the cell nucleus. Tau is also a synaptic protein. Very recently, it has been demonstrated that tau plays a basic role in accelerating spine formation, dendritic elongation, and synaptic plasticity [59]. Native tau modulates NMDA (N-methyl-D-Aspartate) receptor signaling and influences intracellular Ca^2+^ levels. Native tau is a highly soluble protein and not prone to aggregation in its monomeric form. Phosphorylation of tau proteins in specific sites (Thr 231 and Ser 235, 262, 293, 324, and 356) results in the formation of pathological hyperphosphorylated proteins. In a cellular model, Aβ addition was shown to catalyze tau-hyperphosphorylation via activation of protein kinases DYRK1 and Fyn. Hyperphosphorylated tau dissociates from the microtubules and forms toxic assemblies:

abnormally phosphorylated monomer (pTau) → dimer, trimer → small soluble oligomers (oTau) → granular oligomers → straight filaments → paired helical filaments → neurofibrillary tangles.

The monomeric form, filaments, and tangles are probably not toxic; however, the diffusible oligomers are toxic [60]. Recently, several cryo-EM studies have revealed the structure and peptide chain conformation of the helical filaments isolated from an AD brain at the atomic level [61].

Hyperphosphorylated tau aggregates possess several pathophysiological actions:

- Disaggregation and collapse of microtubules. Big tau assemblies may cause a direct physical blockade of axonal transport.
- Loss of DNA protection at the nucleus.
- Increased excitability of neurons.
- Tau may bind to synaptic vesicles and disrupt the synaptic cytoskeleton causing synaptic loss and disturbances of neural circuits.
- Tau causes neuroinflammation.
- Prion-like propagation: tau is able to spread cell to cell, most likely via macropinocytosis and not by receptor support [62]. Several authors propose that AD may be an infectious disease of the brain [63].

It is widely accepted that Aβ and tau proteins have a synergistic effect in the progression of AD: “Aβ is the trigger and tau is the bullet driving AD” [64,65]. It has recently been demonstrated that the presence of both Aβ and tau is necessary for memory decline at the beginning of AD [66]. Aβ and tau crosstalk shows that the two proteins are coupled in the progression of AD.

### 2.4. The Ever-Changing and Developing Amyloid (and Tau) Hypotheses. Alternative Hypotheses of AD

The importance and complexity of AD triggered the elaboration of a very large number of hypotheses dealing with the pathogenesis of the disease. Strong neuropathological and genetic evidence support the mainstream concept of the amyloid hypothesis of AD. According to the hypothesis, an imbalance in the production and clearance of Aβ results in its overproduction and formation of toxic amyloid assemblies. Aβ accumulation and deposition are the critical initial steps, the central events, and driving factors of the progress of AD [67]. Synaptic loss, chronic neuroinflammation, microgliosis, astrocytosis, neuritic dystrophy, tau-hyperphosphorylation, and formation of NFTs are the consequences of amyloid deposits. The hypothesis has been changed several times over the decades. Originally, the amyloid plaques were proposed to be the culprits of the disease [67], followed by hypothesizing such a role for Aβ oligomers [68]. Although inconsistencies and controversies have been observed, the hypothesis has received continuous support for three decades in the field of AD drug development.

Several experimental studies demonstrated that a pool of intracellular Aβ exists in the brain at the very early stage of AD that may interact with subcellular organelles, thereby affecting their normal function [69]. Novel evidence has suggested that extracellular Aβ has a small impact on AD pathology, and the plaques alone cannot be responsible for the whole pathological process [70]. The “intracellular Aβ (iAβ) hypothesis” assumes that accumulation of iAβ in the brain cells is the earliest sign of AD. The toxic iAβ assemblies trigger tau pathology and the formation of NFTs. The formation of extracellular plaques only occurs at later stages of the disease [71,72]. Indeed, a series of experiments have proven the formation and accumulation of iAβ, e.g., observation of iAβ by light and fluorescent microscopy, as well as by EM [73]. iAβ is selectively resistant to enzymic degradation and accumulates in a nonfibrillar form in lysosomes [74]. The release of lysosomal proteases is one of the earliest events of iAβ neurotoxicity [75].

*The tau hypothesis*. Several studies have demonstrated that tau hyperphosphorylation and NFT formation are early events in the development of AD. It was found that pTau cannot bind to the microtubules aggregates to NFTs after truncation of the polypeptide chains [76]. NFTs are toxic and shift APP processing to elevated Aβ production. Experiments demonstrated that pTau is a self-propagating protein with a prionoid character, spreading from cell to cell [77]. The tau (or tau-propagation) hypothesis proposes that pTau dissociates from the microtubules and aggregates to NFTs; this process precedes Aβ plaque formation and drives AD development. It was observed that tau pathology in the brain showed a good correlation with cognitive decline. Moreover, the formation of pTau proved to be the common pathway of different, altered molecular signals [70,78]. According to the tau hypothesis, pTau plays a central role in AD and is the diagnostic and therapeutic target of AD research. Indeed, a shift from the physiological to the pathological level of tau was observed in AD synapses [79].

Most attempts to develop Aβ-targeted drugs for treating AD ended in failure for many years. As a consequence, an inevitable discussion, or rather a debate, started, whether misfolded Aβ or tau amyloids are the culprits, upstream pathogenic causes for driving AD progress. The problems of the amyloid hypothesis in understanding the pathomechanism of LOAD led to a change in the target of drug research from Aβ to tau. However, the experiments with the GSK-3 enzyme inhibitors, such as tideglusib and anti-aggregating agents (methylene blue derivatives: Trx0014 and LMTM), resulted in controversial results, and, thus, did not support the tau hypothesis. As Aβ and tau have neuroprotective effects, it is possible that their overproduction is only a protective response to cellular stress and damage.

Several research groups tried to unify the two hypotheses [79,80,81]. The “dual pathway hypothesis” of Small and Duff tried to reconcile the two hypotheses. Other research groups “revitalized” the tau hypothesis and provided an integrative model of AD pathogenesis [82]. However, the debate has not ended yet. 

The *metal ion hypothesis* connects the two main Alzheimer’s hypotheses. Several transition metal ions participate in physiological processes. They play important roles in the maintenance of brain functions and may regulate the development of AD [83]. Postmortem analysis of amyloid plaques demonstrated the accumulation of copper, iron, and zinc by 5.7, 2.8, and 3.1 times compared to the levels of normal brains, respectively [81]. Heavy metal ions can be bound to the His residues of Aβ (His6, His13, and His14) and to pTau [84]. Metal ion imbalance induces Aβ and tau pathologies [85]. Zinc, copper, and iron ions enhance the production of Aβ and subsequently bind to Aβ and tau, promoting their aggregation. The misbalance of these metal ions is connected to the main factors of the pathogenesis of AD (oxidative stress, protein aggregation, mitochondrial dysfunction, energy deficiency, and neuroinflammation) [86]. Accumulation of iron and copper ions can promote cell death by ferroptosis or cuprostosis [87]. Therefore, metal ion chelators as therapeutic agents have been used for treating AD (Section 3). Metal ion chelators are also potential drug candidates for modulating neuroinflammation in AD.

In addition to the above processes, many other factors can affect the occurrence of AD. The results of novel experiments with rhesus monkeys connect the tau and glutamatergic dysregulation hypothesis [88]. Accordingly, vulnerable glutamatergic neurons are responsible for Ca^2+^-dysregulation, and this event induces the formation of pTau and NFTs. As a consequence, tau pathology might be the key initiating factor for LOAD and suggests that future AD drugs should reduce tau pathology.

The most detailed hypothesis of the initiation and progress of AD was introduced by De Strooper and Karran [89]. The AD continuum includes three stages. First, clearance problems and proteostasis failure lead to abnormal Aβ and tau formation (biochemical phase). Several genes participate in this process (e.g., APOE4, LRP1, ABCA7, SORL 1, PICALM, and AQP4). In the second phase, each type of brain cell (neurons, microglia, astrocytes, oligodendrocytes, the glioneuronal unit, and the neurovascular unit) participates in the progress of degeneration (cellular phase). These changes result in chronic neuroinflammation, chronic imbalances in neuronal circuitry, cell failure, and cell death. The clinical phase is characterized by hippocampal shrinkage, MRI changes in the brain, alterations of CSF, and dementia. The whole process may take two to three decades. 

The history and development of the various AD hypotheses and clinical trials have been excellently reviewed in [90]. The short list of the hypotheses is as follows:

1. Aβ, very probably iAβ, is the initiating factor of AD [66].
2. Loss of cholinergic neurons and neurotransmission are causing factors of AD [91].
3. Deficit of the glutamatergic system [88] triggers tau overproduction.
4. Abnormal phosphorylation of tau proteins is in the background of AD initiation and progress [76].
5. According to the dual cascade hypothesis, cellular processes in the brain cortex simultaneously drive tau and Aβ pathology [80].
6. Metal ion hypothesis: several transition metal ions accelerate amyloid aggregation [84,85].
7. Mitochondrial dysfunction starts a cascade of pathological events in brain cells [92].
8. Chronic neuroinflammation is responsible for the initiation of damage to neurons [93].
9. According to the vascular dysfunction hypothesis, impaired brain circulation and endothelial-mediated processes play central roles in AD pathogenesis [94,95].
10. Impaired amyloid clearance (BBB and glymphatic clearance) is the main cause of amyloid accumulation in AD [96].
11. Aβ peptides are generated in the periphery and enter the brain via the BBB [97].
12. Aging is the main driver of sporadic AD pathogenesis. Each type of brain cell (microglia, astrocytes, and brain vasculature cells) participates in pathophysiological events [89].

**Figure 1:**
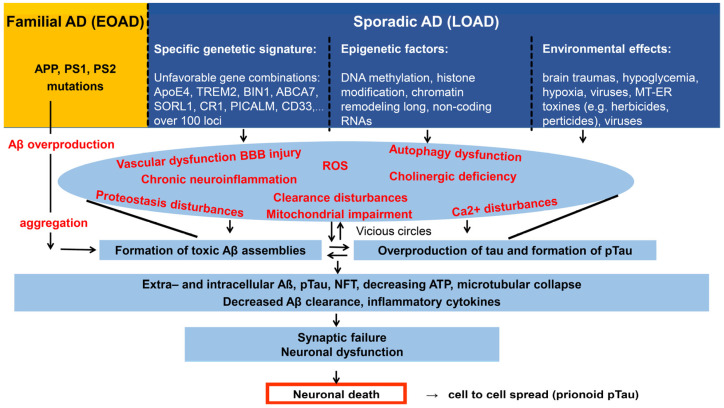
The funnel model of AD. It shows the interrelated destructive processes acting in vicious circles, leading to widespread cell death. In familial AD cases (left side), mutations in APP, presenilin 1 and 2 (PS1 PS2) genes induce Aβ overproduction and formation of toxic aggregates. In the sporadic form (LOAD), aging, unfavorable gene combinations, epigenetic changes, and various environmental factors induce slow changes in the brain. Vascular and autophagy dysfunctions, BBB injury, proteostasis, and clearance disturbances lead to Aβ accumulation and subsequent formation of toxic Aβ and tau assemblies in vicious circles. Microtubular collapse results in synaptic failure, dysfunction, and death of neurons. Aβ and tau act in synergy in the pathological cascades.

We propose that distinct hypotheses might be valid and applicable to understanding the pathology of different forms of AD. In EOAD, the priority of Aβ has already been demonstrated. Well-known mutations of APP, PS1, and PS2 are responsible for the formation of toxic Aβ species, and the familial disease shows autosomal dominant inheritance [98]. The other form of AD, LOAD, is a very heterogeneous disease. It is very probable that not only toxic Aβ can initiate the formation of the “bullet” via tau hyperphosphorylation and NFT formation [64]. Experimental and clinical data have resulted in the consensus that AD is an amyloid-provoked tauopathy. It is agreed that both Aβ and pTau are necessary for cognitive decline [66]. Tau-originated toxic NFT is the common pathway in all subtypes of AD [78]. It can be accepted that either pTau or NFTs alone are able to start AD pathology, e.g., after dysfunction of the glutamatergic system or other stress conditions [88]. However, experimental evidence demonstrates that Aβ (very probably iAβ, which seems to be the earliest sign of pathological events in AD) is the primary factor in starting AD progress in most cases. Figure 1 shows a short summary of the diversity of the possible initiating factors and the pathophysiological processes resulting in cellular dyshomeostasis and cell death.

### 2.5. Early Diagnosis of AD, Molecular Biomarkers

Early diagnosis at the preclinical stages of AD is an important issue since amyloid accumulation in the brain begins more than two decades before cognitive decline. AD continuum means that the progress of cognitive decline has several steps [99]:

chronic stress → subjective cognitive decline (SCD) → mild cognitive impairment (MCI) → AD dementia.

Reversal of AD dementia is not possible yet. Contrary, MCI can be reverted to the normal condition [99]. A very early diagnosis may facilitate preventive pharmacological and nonpharmacological treatments before dementia manifests.

Finding and validating standard clinical diagnostic biomarkers is not an easy task, partly owing to the complexity of AD. The diagnostic process has several subsequent steps from detection of cognitive impairment until treatment (laboratory tests, genotyping, neurological examination, cognitive and functional tests, then brain structural imaging, CSF and blood biomarker analysis, and finally diagnosis) [100]. Prediction of AD progression would be essential for planning treatment and medication. However, it has remained a challenging task so far. It is supposed that AD can be diagnosed based only on biomarker abnormalities [101]. In other words, AD diagnosis can be based purely on biology, without cognitive tests.

*Diagnostic tools* [102]. Brain imaging and fluid biomarker analysis are the most frequently used assays for the detection and staging of the disease. During the last decade, there was a great development in the field of neuroimaging [103]. Precise detection of both Aβ and tau in the blood and brain are essential tools for AD diagnosis. The leading neuroimaging methods have been PET and MRI; less frequently used are CT and SPECT, the latter being a nuclear imaging technique [104]. Functional MRI (fMRI) indirectly measures brain activity and the integrity of brain networks in the MCI stage. Multimodal MRI can be effectively used for the prediction of AD progress [103]. The application and evaluation of different tau biomarkers (tau-PET, CSF- and blood-based markers) have been recently reviewed [105]. The use of second-generation tau-PET tracers improved our understanding of the heterogeneity of AD and helped for staging the disease. The application of brain imaging techniques is reviewed in the World Alzheimer Report 2021 [106].

It is not rare that cognitively normal patients have degeneration in the cholinergic white matter [107,108]. It is assumed that the integrity of cholinergic pathways might be a good indicator of early changes in AD progress. Individuals without cognitive decline but possessing abnormal Aβ and tau PET images (plaques and NFTs) are also at a high risk of AD [109]. When both Aβ and tau are present in the brain, it can no longer be considered a risk factor but rather a diagnosis [105]. Other approaches, such as electroencephalography (EEG), have also been used for early diagnosis of MCI and AD [110].

The levels of fluid biomarkers of AD (mostly Aβ and tau) can be measured in the cerebrospinal fluid (CSF) and blood. CSF analysis has a disadvantage: it needs invasive treatment (lumbar punction). The routine analytical methods are quantitative measurements of Aβ 1–42, pTau 181, and the ratio of pTau 181/Aβ 1–42 by immunoassay [111,112]. CSF concentration of Aβ peptides can also be measured by the automated tandem mass spectrometry method (HPLC-MS/MS) [113]. The U.S. FDA has recently granted marketing approval for a novel CSF-test (Lumipulse G-amyloid ratio(1–42/1–40) as a cheap alternative to PET for early detection of amyloid accumulation in AD brains [114]). (The Aβ 1–42/1–40 ratio in CSF strongly correlates with the PET status of the brain). 

The application of a new blood test, a not complicated binding assay, has recently been published for very early detection of soluble toxic Aβ peptides (SOBA) [115]. Further studies should demonstrate the suitability of the SOBA method for identifying patients at risk of cognitive decline. Parallel work was performed for the detection of Aβ oligomers in mouse brains using a new PET-tracer (a ^64^Cu-labeled aza-peptide) [116]. This approach gave unbelievably early detection of oAβ increase compared to the standard ^11^C-PET method.

A radical improvement in blood tests for AD diagnosis has been developed [117]. It was demonstrated that measuring the levels of multiple blood biomarkers (pTau 231; pTau 231 and Aβ 42/40 together) was sufficient for identifying AD pathology. Blood tests were performed with a group of 242 patients and repeated for up to 6 years, along with MRI and cognitive testing. Evaluation of the experimental data gave interesting results: only pTau 217 was related to typical AD pathology over the 6-year testing period. Thus, pTau 217 may be an ideal biomarker in the clinical phases of AD for monitoring disease progression and the protective effects of drug candidates. “The novel blood test will revolutionize the diagnosis of AD” [117].

## 3. Conventional and Novel Targets for Slowing and/or Preventing the Progress of AD

There are no disease-modifying drugs for AD treatment yet. Existing drugs, e.g., cholinesterase inhibitors (donepezil) and NMDA receptor modulators (memantine), only show a palliative effect. There are serious problems in target identification for the development of AD drugs. Both amyloid proteins (Aβ and tau) possess essential physiological functions in their native state. Therefore, the full blockade of their biosynthesis may cause very severe side effects [118]. The molecular heterogeneity of Aβ and tau assemblies makes target identification very problematic: which species should be targeted to stop neurodegeneration? If aging is the most dangerous risk factor for AD, how can we slow down the natural aging processes? Finally, the lack of a good animal model for AD causes difficulties in translating the results of animal experiments. 

Various biochemical pathways that suppress or remove aggregated proteins are now targeted and examined after a series of failed clinical studies with old AD-drug candidates. Several of these pathways of AD are summarized in Figure 1. The application of multitarget Alzheimer’s drugs has very recently been widely reviewed [119]. Intracellular Aβ is also a therapeutic target [120]. 

### 3.1. Inhibition of the Formation of Toxic Amyloid Aggregates

#### 3.1.1. Decreasing Aβ Production

The first approaches to AD drug development were focused on the partial inhibition of the formation Aβ peptides. Inhibitors of γ- and β-secretases, the key enzymes of amyloidogenic APP processing, have reached phase 2 and phase 3 steps in clinical trials. Although β-Secretase 1 (BACE 1) inhibitors reduced Aβ production, the drug candidates were not able to slow down cognitive decline [118]. γ-Secretase inhibitors (e.g., avagacestat and semagacestat) caused cognitive loss and other severe side effects.

Activation of the nonamyloidogenic pathway of APP procession by activation of α-secretase might be another approach for decreasing Aβ biosynthesis. Unfortunately, the α-secretase enzyme activators also gave discouraging results in the clinical application [121].

#### 3.1.2. Blocking Aβ Aggregation

The mechanism of amyloid protein aggregation has been recently reviewed [122,123,124]. Targeting Aβ aggregation is a very frequently used strategy in AD drug development [125]. Many natural or synthetic small molecules, peptides, and peptidomimetics were studied for their modulatory effect on Aβ aggregation [118]. Polyphenols, tetracyclines, anthracyclines, and sterols have been used as anti-amyloid compounds [125]. 

The neuroprotective effect of several natural polyphenols (resveratrol, epigallocatechin, epigallocatechin-3-gallate (EGCG), myricetin, curcumin, and quercetin) has been studied [126]. Myricetin inhibits Aβ nucleation, while resveratrol, curcumin, quercetin, and EGCG inhibit fibril elongation. Resveratrol and curcumin also decrease the hyperphosphorylation and aggregation of tau protein. Most of the polyphenols (besides inhibiting amyloid aggregation) have an antioxidant effect scavenging free oxygen radicals and protecting DNA from oxidative damage. Resveratrol also modulates neuroinflammation and induces adaptive immunity. Unfortunately, the bioavailability of resveratrol and curcumin is very low. Recently resveratrol was combined with selenium nanoparticles (formation of ResSeNPs). The novel combination has good absorption and might improve resveratrol application in AD treatment [127]. Chitosan-containing selenium nanoparticles (Ch-SeNPs) also inhibited D-amino acid enantiomeric-induced amyloid aggregation [46].

Multifunctional metal chelators of the different chemical structures have been designed as potential anti-AD drugs as metal dyshomeostasis contributes to the onset and progression of neurodegeneration [128,129]. Clioquinol, a metal-protein attenuating compound, chelates copper and zinc ions and decreases amyloid aggregation in the brain [130]. PBT2, a clioquinol-related second-generation drug, has been used in clinical studies for the treatment of AD. The Phase 2 study in 42 prodromal AD or mild AD patients gave negative results. However, novel metal chelators might have a better chance. Recent studies with a BBB-permeable silica-cyclen nanochelator gave positive results in a cellular assay [131].

Native state stabilization in a highly crowded cellular environment [124] seems to be a novel method of choice for inhibiting the formation of toxic Aβ aggregates. Peptides designed for mimicking the Aβ amyloid core (ACM-peptides) coaggregate with Aβ 1–42 and form nontoxic nanofibers. Proteases easily degrade these fibers, and thus, ACMs might be ideal anti-amyloid drug candidates ([132]. On the contrary, medin, a known protein, promotes the formation of amyloid aggregates and deposits by coaggregation with Aβ. Researchers hope that medin inhibitors could block the formation of amyloid deposits and may behave as anti-amyloid agents [133]. It has been published very recently that 14-3-3 proteins also bind amyloids, and thus, they can also be targeted as an anti-aggregation approach [134].

#### 3.1.3. Blocking Tau Biosynthesis 

Enzyme inhibitors: Tideglusib, a selective inhibitor of the tau-phosphorylating enzyme GSK-3β, was planned to reduce tau-hyperphosphorylation, leading to pTau aggregation and propagation. Tideglusib and similar compounds were able to reduce the amyloid formation and neuroinflammation. However, the clinical trials have not shown any benefits [118]. 

#### 3.1.4. Inhibiting Tau Aggregation and Fibrillation

Small molecules, such as a leukomethylene blue derivative (LMTM), have been used as a tau aggregation inhibitor. The treatment was unsuccessful in a group of 800 patients with mild AD in phase 3 clinical trials [135]. Several compounds have been recently studied for inhibiting tau-fibrillation, till now without success [123].

#### 3.1.5. Other Tau-Directed Potential Approaches

Inhibition of the retromer complex, a cargo-sorting protein assembly, increased the toxicity of human tau via increased tau-truncation in an animal model [136]. Retromer deficiency was also connected with tau pathology in Down syndrome patients [137]. There is hope that these results can be translated into AD drug design. Bassoon (BSN), a synaptic scaffold protein, also has an interesting effect: it interacts with tau seeds and contributes to tau propagation and neurotoxicity [138]. In mice, BSN downregulation reduced pathology and propagation by decreasing the stability of tau seeds. The inhibition of BSN-tau interactions might be a novel therapeutic approach for treating AD and other tauopathies.

### 3.2. Improvement of Amyloid Clearance. Vascular Dysfunction, BBB, and the Glymphatic System

Increased concentration of soluble Aβ (sAβ) in the brain is highly correlative with the severity of neurodegeneration [139], as sAβ seems to be responsible for the deterioration of synaptic function [140]. Two processes contribute to Aβ accumulation in the brain: Aβ production by APP cleavage and transport via the BBB.

#### 3.2.1. Passive Immunotherapy with Monoclonal Antibodies (mAbs)

Passive immunotherapy uses exogenous humanized monoclonal or for the promotion of Aβ clearance from the brain ([141,142]. Several mAbs (Bapineuzumab, Gantenerumab, Aducanumab, Donanemab, Solanezumab, and Crenezumab) have been developed for targeting amyloid plaques against different epitope regions of Aβ [143]. Recently, six mAbs have entered phase 3 trials, and Aducanumab (Biogen) was approved by the U.S. FDA for marketing [142]. However, the European Medicine Agency (EMA) refused the approval of Aducanumab, and it is not authorized in Europe. Aducanumab got approval for 4 years for performing a postapproval confirmatory trial (phase 4 trial). As for the neuroprotective action of humanized mAbs, they target amyloid plaques and not monomers. The main therapeutic hypothesis for the application of hmAbs is that reduction in Aβ and clearance of amyloid plaques would be required for restoring homeostasis in the brain. Aducanumab showed a robust decrease in amyloid burden and full removal of amyloid plaques. Unfortunately, the drug shows a dose-related adverse effect: one-third of the patients showed “amyloid-related imaging abnormalities” (ARIAs) that can be fatal.

Lecanemab (Eisai) treatment, designed to neutralize toxic Aβ protofibrils, has given the best results in clinical studies till now. The phase 3 Clarity trial was performed with 1800 individuals (10 mg/kg mAb infusion every two weeks, 18 months study with early AD patients). This treatment resulted in promising changes in the brain (reduced Aβ and tau scan). However, cellular death was unaffected, and slowing the progress of dementia was modest (only a 0.45 score on the cognition rating scale) [144]. Bleeding in the brain as a potential risk of the lecanemab treatment was present in 22% of patients. The pharmaceutical enterprise Eisai applied for accelerated approval of the drug to the U.S. FDA, and the decision on January 6th this year was “full approval”. The success of lecanemab was a big step in AD drug research but initiated a hot debate owing to the modest effect and the risks of application.

#### 3.2.2. Clearance by the BBB and Activation of the Glymphatic System 

Dysfunction of the neurovascular unit (NVU) may play a decisive role in AD progress [95]. NVU is a complex system of pial arteries, penetrating arterioles, intraparenchymal arteries, and small capillaries. BBB is a specific brain composition of endothelial cells in NVU, together with pericytes, basement membrane, and astroglial end feet. NVU dysfunction is an essential element of AD pathogenesis and a molecular target for AD therapies by prevention and repair of NVU damage. 

Age-related declining efficacy of BBB/proteolytic pathways causes a decrease in CSF-based Aβ clearance in humans [96].

In the late stage of AD patients, the production of Aβ peptides is relatively constant. However, Aβ clearance is impaired. Brain capillary endothelial cells mediate Aβ clearance, leading to Aβ accumulation in the brain. Aβ binding transport proteins, e.g., ApoE and clusterin, as well as receptors (e.g., low-density lipoprotein receptor (LRP1), the receptor for advanced glycosylation end product (RAGE), P-glycoprotein transporter (Pgp or ABCB1)) are expressed in BBB and control Aβ efflux and influx across the BBB.

Aβ deteriorates the components of NVU, and it induces endothelial cell injury by promoting free radical (reactive oxygen species—ROS and reactive nitrogen species—RNS) formation. Deposition of Aβ in BBB contributes to the damage of BBB [145]. Aβ induces the release of inflammatory cytokines and chemokines (that leads to chronic neuroinflammation), impairs BBB, and decreases blood flow by up to 40%. Endothelial cell receptors and transporters may serve as potential therapeutic targets for increasing Aβ clearance [146]. Reduction in RAGE activity and upregulation of LRP1 and Pgp expression will increase Aβ clearance from the brain [146]. Endothelins (ETs, distinctive peptides of 21 amino acids) are well-known vasoconstrictive agents [147]. Endothelin is a novel target of AD drug research: ET-receptor agonists and antagonists proved to be effective for the prevention of AD in preclinical studies [147].

The glymphatic system (GS) is a unique fluid-transport pathway in the brain as it provides access to all brain regions. It uses the network of tunnels created by astrocytes, the interstitial space between cells such as the lymphatic system. GS pathway plays an important role in cleaning waste from the brain [148]. If the GS does not work properly, toxic waste (e.g., Aβ and pTau peptides) is not removed from the brain. ApoE plays a decisive role in removing Aβ assemblies by the glymphatic system. Epigenetic changes (reduced methylation of the APOE gene) are linked to AD progress. Aquaporin 4 (AQP4) water channel is also an important factor for the GS: the loss of polarity of AQP4 reduces GS functions. BBB and the GS interact in solute waste clearance in the late stages of AD [149].

GS fluid transport is suppressed in acute and chronic neuroinflammation [150]. Modulation of brain fluid transport may be a novel target for developing new drugs to fight acute and chronic inflammation in the brain. There is evidence that the presence of increased wasteosomes (or corpora amylacea i.e., amyloid bodies) is an indicator of chronic failure of the GS activity [151]. Chronic lymphatic insufficiency is a risk factor for NDDs, and thus, improvement of the GS is a novel target of AD drug strategies.

### 3.3. Modulation of Chronic Neuroinflammation

#### 3.3.1. Chronic Neuroinflammation

Chronic neuroinflammation (NI) begins in the second phase of AD progress, in the cellular phase [89]. NI is a protective response against different factors causing CNS damage. NI plays an essential role in AD pathogenesis. The innate immune system represents the first line of defense. It was published in 2015 that chronic comorbidities (e.g., arthritis, atherosclerosis, obesity, and metabolic diseases), as well as low-grade systemic inflammation, are risk factors for subsequent dementia [152]. IL-1β, TNF-α, and other proinflammatory mediators may drive hypothalamic dysfunction, impair neurogenesis, and cause cognitive functional decline [93,152]. Cytokine and chemokine signaling play a decisive role in inflammatory diseases, and therefore, they can drive the processes of AD development [153]. Neuroinflammatory markers [154,155] are very important indicators of pathological processes of NDDs [154]. External pathogens activate the brain’s innate immune system (e.g., microglia and astrocytes) for the protection of brain cells. However, overactivation of the immune cells results in the release of proinflammatory factors (TNF-α, IL-1β, IL-18, NO, and others). Neuroinflammation begins in the cellular phase of AD and is a potential risk for dementia [156,157]. Soluble oAβ can also activate microglia, producing inflammatory cytokines, besides external pathogens [158]. Aβ activates several microglial receptors, such as CD36, and thereby, it triggers the secretion of proinflammatory cytokines, chemokines, and ROS.

Aβ assemblies can trigger the formation of the inflammasome NLRP3, lysosomal disruption, and release of cathepsin-B [158]. Inflammasomes are sensors and regulators of cellular injury and inflammation. Chronic triggering of inflammasomes in the brain tissue leads to an increased level of IL-18 family cytokines [159]. Autophagy dysfunction may be associated with AD pathology, and autophagy activation may suppress neuroinflammation by degrading inflammasomes [160]. The development of small molecular autophagy inducers represents a pharmacological opportunity for the protection of neurons in AD [161].

Microglia (MG) are central players in AD progress [162]. MG has an essential task in the brain which is the perpetual active surveillance of synapses. Since MG are phagocytic cells, they clean the brain tissue from terminally injured neurons, cellular corpses, and debris [163]. MG also has a decisive role in the maturation of the brain by phagocytosis. Deteriorated senescent neurons may cause NI in the AD brain if microglial cleanup does not work properly [164]. Impairment of normal microglial function causes serious effects on normal brain development. MG also regulates myelin growth and integrity in the CNS [165]. MG interacts with neurons and modulates neuronal activity [166]. Absence of MG results in significantly increased brain injury [167]. GWAS studies demonstrated that many of the LOAD risk genes are mainly expressed in MG and not in neurons [168]. “Different AD risk factors converge on the activation response of microglia” [27,28,29,169].

MG has a mesodermal origin and myeloid nature [170]. Physiological and pathological forms of MG have remarkable morphological diversity. Interleukins activate MG resulting in morphological changes and upregulation of many pro- and anti-inflammatory cytokines. There are several forms of MG: (1) resting and quiescent, homeostatic MG with ramified morphology, (2) disease-associated, pro-phagocytic MG, and (3) dystrophic or primed, aberrant MG, deramified with shortening and loss of branches [171]. Drug researchers should find a suitable form of MG that could serve as a target for neuroprotection.

Astrocytes are the other important immune cells that participate in the neuroinflammatory process and maintain plasticity in the adult brain. Astrocytes serve as targets of future AD drugs [172]. 

#### 3.3.2. Neuroinflammation and Glial Cells as Targets for AD Drug Development

Modulating neuroinflammation and the activity of glial cells are novel targets in AD research (reviewed in [172,173]). Therapeutic strategies include the use of different anti-inflammatory drugs (e.g., indomethacin, VX-745, OTI-125, candesartan, celecoxib, etanercept, and atomoxetin) in different clinical phases. The newest clinical trials are summarized in [172]. Five experiments are in phase 1, nine in phase 2, and one in phase 3 trial. The following potential drug targets have been identified:

1. TNFα modulation [174].
2. Activation of spleen tyrosine kinase (SYK) for increasing the clearance function of MG [175].
3. Activation of the CX3CR1 (C-X3-C Motif Chemokine Receptor 1) gene for improved MG phagocytotic activity [176].
4. Activation of TREM2 microglial protein for slowing down pTau accumulation and cognitive loss [177].
5. Activation of the PLCG (Phospholipase C γ1) enzyme for promoting the protective function of microglia [178].
6. Mitigation of neuroinflammation by increasing the scavenger functions of MG by targeting INPP5 (Inositol Polyphosphate-5-Phosphatase) enzyme [179].
7. Modulation of astrocyte activity for Aβ degradation and clearance, as well as BBB protection [180].
8. Creating antioxidant molecules for protecting the brain from the degrading effect of ROS by activation of mucosal-associated invariant T-cells (MAIT-cells) [181].
9. Lowering or removing ApoE4 from neurons for decreasing aberrant microglia activation [27,28,29]

The results of future preclinical and clinical studies will decide which targets prove to be successful in AD drug development.

## 4. Conclusions and Outlook

An old dream has been to find a master gene or gene combination whose modulation would slow down the aging process, thereby helping to avoid age-related dementia. This type of master gene has not been found yet. AD drug research has reached a turning point now. The novel targets for biomarker and drug research are not amyloid plaques but intracellular proteins (iAβ and oTau) or biochemical pathways. The newest approaches of precise fluid biomarker determinations by cheap clinical mass spectrometry provide a means for the diagnosis of MCI and AD in a large population. It has been widely accepted in recent years that both amyloids (Aβ and pTau assemblies) participate in the progress of AD. In addition, a drug combination ought to be used for reversing MCI or slowing down the development of AD. Novel protein targets (e.g., medin, BSN, PAR-5) should be used for the prevention of amyloid overproduction and the formation of toxic assemblies. Small molecular inducers of small, neuroprotective Hsps (e.g., crystallines: Hsp B4/5) will be used for inhibiting the formation of toxic amyloid aggregates. Increased Aβ clearance with the help of monoclonal Abs might be reached by increasing the penetration of big proteins across the BBB and the cell membrane by specific transporters. Special drugs may be developed to augment the effectivity of oAβ and oTau clearance by the BBB and the glymphatic system from the aging brain. The dangerous effect of chronic inflammation on neuronal death might be inhibited via novel anti-inflammatory agents (e.g., Sig-1R ligands). Microglia, a double-edged sword in the maintenance of brain homeostasis in a suitable form, might be ideal for phagocytotic clearance of brain waste. Identification of the biochemical markers of activated microglia and the main mediators of neuroinflammation provide novel methods for the selective manipulation of microglia in the future. Novel approaches using rejuvenated immune cells and glial phagocytosis by astrocyte-like Bergman glial cells might also increase Aβ clearance. Regulation of ApoE4 effect on cholesterol transport and myelination may help AD patients who have a special ApoE4 gene signature. It is expected that physical methods, e.g., transcranial magnetic stimulation, may also prove to be successful in treating AD.

Currently, there are over 120 different potential new medications for AD in clinical trials. We conclude that over 30 years of intensive experimental work has not provided a genuine breakthrough in AD treatment. A good and suitable animal model of AD for preclinical experiments would be needed. Development of such kind of rodent model mimicking the changes during aging (BBB dysfunction, decreasing Aβ clearance, Aβ and tau overproduction, and NI) could be reached by transgenic techniques [182]. The efficacy of Aβ binding mAbs will be improved during the next years, but their disadvantage is that they only target the extracellular amyloid plaques. The spectrum of drug targets should be widened. 

The development of small molecular oral agents for intracellular targets and biological processes (amyloid aggregation, clearance, Hsp, autophagy induction, inflammasomes, etc.) will be the focus of future AD drug research. The development of β- and γ-secretase inhibitors will be abandoned. In the near future, the selection of endangered individuals using genome sequencing and the identification of bad genetic signatures may become feasible. The earliest possible diagnosis (latest in MCI stage) will be necessary for successful AD treatment as aging is the most important risk factor of AD. We suggest that a single drug cannot be expected to treat all stages of AD. A drug combination strategy with multiple molecular targets should be considered. It may take a couple of years to developing successful AD treatments.

## Data Availability

Data sharing is not applicable to this article.

## Abbreviations

APP: amyloid precursor protein, AQP4: aquaporin-4, BSN: bassoon protein, CSF: cerebrospinal fluid, GS: glymphatic system, mAB: monoclonal antibody, MG: microglia, MT: microtubules, NDD: neurodegenerative disease, NI: neuroinflammation, NVU: neurovascular system, and sAβ: soluble Aβ.

## References

[1] Vaquer-Alicea J., Diamond M.I. (2019). Propagation of Protein Aggregation in Neurodegenerative Diseases. Annu. Rev. Biochem..

[2] Taylor A.I.P., Staniforth R.A. (2022). General Principles Underpinning Amyloid Structure. Front. Neurosci..

[3] Bogár F., Fülöp L., Penke B. (2022). Novel Therapeutic Target for Prevention of Neurodegenerative Diseases: Modulation of Neuroinflammation with Sig-1R Ligands. Biomolecules.

[4] Small G.W. (2022). Updates in the Management of Mild Cognitive Impairment and Alzheimer Disease. J. Fam. Pract..

[5] Ju Y., Tam K. (2022). Pathological Mechanisms and Therapeutic Strategies for Alzheimer’s Disease. Neural Regen. Res..

[6] Kovacs G. (2016). Molecular Pathological Classification of Neurodegenerative Diseases: Turning towards Precision Medicine. Int. J. Mol. Sci..

[7] Rahimi J., Kovacs G.G. (2014). Prevalence of Mixed Pathologies in the Aging Brain. Alzheimers Res. Ther..

[8] Trejo-Lopez J.A., Yachnis A.T., Prokop S. (2022). Neuropathology of Alzheimer’s Disease. Neurotherapeutics.

[9] Jellinger K.A. (2022). Recent Update on the Heterogeneity of the Alzheimer’s Disease Spectrum. J. Neural Transm..

[10] Thal D.R., Capetillo-Zarate E., Del Tredici K., Braak H. (2006). The Development of Amyloid Beta Protein Deposits in the Aged Brain. Sci. Aging Knowl. Environ..

[11] Hojjati S.H., Feiz F., Ozoria S., Razlighi Q.R. (2021). Alzheimer’s Disease Neuroimaging Initiative Topographical Overlapping of the Amyloid-β and Tau Pathologies in the Default Mode Network Predicts Alzheimer’s Disease with Higher Specificity. J. Alzheimers Dis. JAD.

[12] Braak H., Braak E. (1991). Neuropathological Stageing of Alzheimer-Related Changes. Acta Neuropathol..

[13] Thal D.R., Rüb U., Orantes M., Braak H. (2002). Phases of Aβ-Deposition in the Human Brain and Its Relevance for the Development of AD. Neurology.

[14] Lee J., Kim H.-J. (2022). Normal Aging Induces Changes in the Brain and Neurodegeneration Progress: Review of the Structural, Biochemical, Metabolic, Cellular, and Molecular Changes. Front. Aging Neurosci..

[15] Livingston G., Huntley J., Sommerlad A., Ames D., Ballard C., Banerjee S., Brayne C., Burns A., Cohen-Mansfield J., Cooper C. (2020). Dementia Prevention, Intervention, and Care: 2020 Report of the Lancet Commission. Lancet.

[16] Lindner A.B., Demarez A. (2009). Protein Aggregation as a Paradigm of Aging. Biochim. Biophys. Acta BBA Gen. Subj..

[17] Groh N., Bühler A., Huang C., Li K.W., van Nierop P., Smit A.B., Fändrich M., Baumann F., David D.C. (2017). Age-Dependent Protein Aggregation Initiates Amyloid-β Aggregation. Front. Aging Neurosci..

[18] Kaushik S., Cuervo A.M. (2015). Proteostasis and Aging. Nat. Med..

[19] Freer R., Sormanni P., Vecchi G., Ciryam P., Dobson C.M., Vendruscolo M. (2016). A Protein Homeostasis Signature in Healthy Brains Recapitulates Tissue Vulnerability to Alzheimer’s Disease. Sci. Adv..

[20] Roher A.E., Lowenson J.D., Clarke S., Wolkow C., Wang R., Cotter R.J., Reardon I.M., Zürcher-Neely H.A., Heinrikson R.L., Ball M.J. (1993). Structural Alterations in the Peptide Backbone of Beta-Amyloid Core Protein May Account for Its Deposition and Stability in Alzheimer’s Disease. J. Biol. Chem..

[21] Lambeth T.R., Riggs D.L., Talbert L.E., Tang J., Coburn E., Kang A.S., Noll J., Augello C., Ford B.D., Julian R.R. (2019). Spontaneous Isomerization of Long-Lived Proteins Provides a Molecular Mechanism for the Lysosomal Failure Observed in Alzheimer’s Disease. ACS Cent. Sci..

[22] Geiger T., Clarke S. (1987). Deamidation, Isomerization, and Racemization at Asparaginyl and Aspartyl Residues in Peptides. Succinimide-Linked Reactions That Contribute to Protein Degradation. J. Biol. Chem..

[23] Truscott R.J.W., Schey K.L., Friedrich M.G. (2016). Old Proteins in Man: A Field in Its Infancy. Trends Biochem. Sci..

[24] Neuner S.M., Tcw J., Goate A.M. (2020). Genetic Architecture of Alzheimer’s Disease. Neurobiol. Dis..

[25] Jonsson T., Atwal J.K., Steinberg S., Snaedal J., Jonsson P.V., Bjornsson S., Stefansson H., Sulem P., Gudbjartsson D., Maloney J. (2012). A Mutation in APP Protects against Alzheimer’s Disease and Age-Related Cognitive Decline. Nature.

[26] Sims R., Hill M., Williams J. (2020). The Multiplex Model of the Genetics of Alzheimer’s Disease. Nat. Neurosci..

[27] Shi Y., Yamada K., Liddelow S.A., Smith S.T., Zhao L., Luo W., Tsai R.M., Spina S., Grinberg L.T., Rojas J.C. (2017). ApoE4 Markedly Exacerbates Tau-Mediated Neurodegeneration in a Mouse Model of Tauopathy. Nature.

[28] Koutsodendris N., Blumenfeld J., Agrawal A., Traglia M., Grone B., Zilberter M., Yip O., Rao A., Nelson M.R., Hao Y. Neuronal APOE4 Removal Protects against Tau-Mediated Gliosis, Neurodegeneration and Myelin Deficits. Nat. Aging.

[29] Shi Y., Manis M., Long J., Wang K., Sullivan P.M., Remolina Serrano J., Hoyle R., Holtzman D.M. (2019). Microglia Drive APOE-Dependent Neurodegeneration in a Tauopathy Mouse Model. J. Exp. Med..

[30] Yeh F.L., Hansen D.V., Sheng M. (2017). TREM2, Microglia, and Neurodegenerative Diseases. Trends Mol. Med..

[31] Bellenguez C., Grenier-Boley B., Lambert J.-C. (2020). Genetics of Alzheimer’s Disease: Where We Are, and Where We Are Going. Curr. Opin. Neurobiol..

[32] Bellenguez C., Küçükali F., Jansen I.E., Kleineidam L., Moreno-Grau S., Amin N., Naj A.C., Campos-Martin R., Grenier-Boley B., Andrade V. (2022). New Insights into the Genetic Etiology of Alzheimer’s Disease and Related Dementias. Nat. Genet..

[33] Li Y., Laws S.M., Miles L.A., Wiley J.S., Huang X., Masters C.L., Gu B.J. (2021). Genomics of Alzheimer’s Disease Implicates the Innate and Adaptive Immune Systems. Cell Mol. Life Sci..

[34] Maity S., Farrell K., Navabpour S., Narayanan S.N., Jarome T.J. (2021). Epigenetic Mechanisms in Memory and Cognitive Decline Associated with Aging and Alzheimer’s Disease. Int. J. Mol. Sci..

[35] Penke B., Bogár F., Fülöp L. (2017). β-Amyloid and the Pathomechanisms of Alzheimer’s Disease: A Comprehensive View. Molecules.

[36] Penke B., Bogár F., Paragi G., Gera J., Fülöp L. (2019). Key Peptides and Proteins in Alzheimer’s Disease. Curr. Protein Pept. Sci..

[37] Jeong H., Shin H., Hong S., Kim Y. (2022). Physiological Roles of Monomeric Amyloid-β and Implications for Alzheimer’s Disease Therapeutics. Exp. Neurobiol..

[38] Nichols R.A., Gulisano W., Puzzo D. (2022). Editorial: Beta Amyloid: From Physiology to Pathogenesis. Front. Mol. Neurosci..

[39] Kent S.A., Spires-Jones T.L., Durrant C.S. (2020). The Physiological Roles of Tau and Aβ: Implications for Alzheimer’s Disease Pathology and Therapeutics. Acta Neuropathol..

[40] Mohamed Asik R., Suganthy N., Aarifa M.A., Kumar A., Szigeti K., Mathe D., Gulyás B., Archunan G., Padmanabhan P. (2021). Alzheimer’s Disease: A Molecular View of β-Amyloid Induced Morbific Events. Biomedicines.

[41] Penke B., Szűcs M., Bogár F. (2020). Oligomerization and Conformational Change Turn Monomeric β-Amyloid and Tau Proteins Toxic: Their Role in Alzheimer’s Pathogenesis. Molecules.

[42] Diociaiuti M., Bonanni R., Cariati I., Frank C., D’Arcangelo G. (2021). Amyloid Prefibrillar Oligomers: The Surprising Commonalities in Their Structure and Activity. Int. J. Mol. Sci..

[43] Owen M.C., Gnutt D., Gao M., Wärmländer S.K.T.S., Jarvet J., Gräslund A., Winter R., Ebbinghaus S., Strodel B. (2019). Effects of in Vivo Conditions on Amyloid Aggregation. Chem. Soc. Rev..

[44] Ivanova M.I., Lin Y., Lee Y.-H., Zheng J., Ramamoorthy A. (2021). Biophysical Processes Underlying Cross-Seeding in Amyloid Aggregation and Implications in Amyloid Pathology. Biophys. Chem..

[45] Srivastava A.K., Pittman J.M., Zerweck J., Venkata B.S., Moore P.C., Sachleben J.R., Meredith S.C. (2019). Β-Amyloid Aggregation and Heterogeneous Nucleation. Protein Sci..

[46] Vicente-Zurdo D., Rodríguez-Blázquez S., Gómez-Mejía E., Rosales-Conrado N., León-González M.E., Madrid Y. (2022). Neuroprotective Activity of Selenium Nanoparticles against the Effect of Amino Acid Enantiomers in Alzheimer’s Disease. Anal. Bioanal. Chem..

[47] Ono K., Watanabe-Nakayama T. (2021). Aggregation and Structure of Amyloid β-Protein. Neurochem. Int..

[48] Lutter L., Al-Hilaly Y.K., Serpell C.J., Tuite M.F., Wischik C.M., Serpell L.C., Xue W.-F. (2022). Structural Identification of Individual Helical Amyloid Filaments by Integration of Cryo-Electron Microscopy-Derived Maps in Comparative Morphometric Atomic Force Microscopy Image Analysis. J. Mol. Biol..

[49] Willbold D., Strodel B., Schröder G.F., Hoyer W., Heise H. (2021). Amyloid-Type Protein Aggregation and Prion-like Properties of Amyloids. Chem. Rev..

[50] Lövestam S., Scheres S.H.W. (2022). High-Throughput Cryo-EM Structure Determination of Amyloids. Faraday Discuss..

[51] Yang Y., Arseni D., Zhang W., Huang M., Lövestam S., Schweighauser M., Kotecha A., Murzin A.G., Peak-Chew S.Y., Macdonald J. (2022). Cryo-EM Structures of Amyloid-β 42 Filaments from Human Brains. Science.

[52] Nishitsuji K., Tomiyama T., Ishibashi K., Ito K., Teraoka R., Lambert M.P., Klein W.L., Mori H. (2009). The E693Δ Mutation in Amyloid Precursor Protein Increases Intracellular Accumulation of Amyloid β Oligomers and Causes Endoplasmic Reticulum Stress-Induced Apoptosis in Cultured Cells. Am. J. Pathol..

[53] Tomiyama T., Matsuyama S., Iso H., Umeda T., Takuma H., Ohnishi K., Ishibashi K., Teraoka R., Sakama N., Yamashita T. (2010). A Mouse Model of Amyloid Oligomers: Their Contribution to Synaptic Alteration, Abnormal Tau Phosphorylation, Glial Activation, and Neuronal Loss In Vivo. J. Neurosci..

[54] Cline E.N., Bicca M.A., Viola K.L., Klein W.L. (2018). The Amyloid-β Oligomer Hypothesis: Beginning of the Third Decade. J. Alzheimers Dis..

[55] Arakhamia T., Lee C.E., Carlomagno Y., Duong D.M., Kundinger S.R., Wang K., Williams D., DeTure M., Dickson D.W., Cook C.N. (2020). Posttranslational Modifications Mediate the Structural Diversity of Tauopathy Strains. Cell.

[56] Tapia-Rojas C., Cabezas-Opazo F., Deaton C.A., Vergara E.H., Johnson G.V.W., Quintanilla R.A. (2019). It’s All about Tau. Prog. Neurobiol..

[57] Sexton C., Snyder H., Beher D., Boxer A.L., Brannelly P., Brion J., Buée L., Cacace A.M., Chételat G., Citron M. (2022). Current Directions in Tau Research: Highlights from Tau 2020. Alzheimers Dement..

[58] Naseri N.N., Wang H., Guo J., Sharma M., Luo W. (2019). The Complexity of Tau in Alzheimer’s Disease. Neurosci. Lett..

[59] Robbins M., Clayton E., Kaminski Schierle G.S. (2021). Synaptic Tau: A Pathological or Physiological Phenomenon?. Acta Neuropathol. Commun..

[60] Shafiei S.S., Guerrero-Muñoz M.J., Castillo-Carranza D.L. (2017). Tau Oligomers: Cytotoxicity, Propagation, and Mitochondrial Damage. Front. Aging Neurosci..

[61] Fitzpatrick A.W.P., Falcon B., He S., Murzin A.G., Murshudov G., Garringer H.J., Crowther R.A., Ghetti B., Goedert M., Scheres S.H.W. (2017). Cryo-EM Structures of Tau Filaments from Alzheimer’s Disease. Nature.

[62] Zhang H., Cao Y., Ma L., Wei Y., Li H. (2021). Possible Mechanisms of Tau Spread and Toxicity in Alzheimer’s Disease. Front. Cell Dev. Biol..

[63] Vasili E., Dominguez-Meijide A., Outeiro T.F. (2019). Spreading of α-Synuclein and Tau: A Systematic Comparison of the Mechanisms Involved. Front. Mol. Neurosci..

[64] Roda A., Serra-Mir G., Montoliu-Gaya L., Tiessler L., Villegas S. (2022). Amyloid-Beta Peptide and Tau Protein Crosstalk in Alzheimer’s Disease. Neural Regen. Res..

[65] Bloom G.S. (2014). Amyloid-β and Tau: The Trigger and Bullet in Alzheimer Disease Pathogenesis. JAMA Neurol..

[66] Sperling R.A., Mormino E.C., Schultz A.P., Betensky R.A., Papp K.V., Amariglio R.E., Hanseeuw B.J., Buckley R., Chhatwal J., Hedden T. (2019). The Impact of Amyloid-Beta and Tau on Prospective Cognitive Decline in Older Individuals. Ann. Neurol..

[67] Hardy J., Allsop D. (1991). Amyloid Deposition as the Central Event in the Aetiology of Alzheimer’s Disease. Trends Pharmacol. Sci..

[68] Selkoe D.J., Hardy J. (2016). The Amyloid Hypothesis of Alzheimer’s Disease at 25 Years. EMBO Mol. Med..

[69] Penke B., Tóth A.M., Földi I., Szűcs M., Janáky T. (2012). Intraneuronal β-Amyloid and Its Interactions with Proteins and Subcellular Organelles: Proteomics and 2DE. ELECTROPHORESIS.

[70] Morris G.P., Clark I.A., Vissel B. (2014). Inconsistencies and Controversies Surrounding the Amyloid Hypothesis of Alzheimer’s Disease. Acta Neuropathol. Commun..

[71] Hartmann T. (1999). Intracellular Biology of Alzheimer’s Disease Amyloid Beta Peptide. Eur. Arch. Psychiatry Clin. Neurosci..

[72] Friedrich R.P., Tepper K., Rönicke R., Soom M., Westermann M., Reymann K., Kaether C., Fändrich M. (2010). Mechanism of Amyloid Plaque Formation Suggests an Intracellular Basis of Aβ Pathogenicity. Proc. Natl. Acad. Sci. USA.

[73] Takahashi R.H., Nagao T., Gouras G.K. (2017). Plaque Formation and the Intraneuronal Accumulation of β-Amyloid in Alzheimer’s Disease: Intraneuronal Accumulation of β-Amyloid. Pathol. Int..

[74] Glabe C. (2001). Intracellular Mechanisms of Amyloid Accumulation and Pathogenesis in Alzheimer’s Disease. J. Mol. Neurosci..

[75] Ditaranto K., Tekirian T.L., Yang A.J. (2001). Lysosomal Membrane Damage in Soluble Aβ-Mediated Cell Death in Alzheimer’s Disease. Neurobiol. Dis..

[76] Grundke-Iqbal I., Iqbal K., Tung Y.C., Quinlan M., Wisniewski H.M., Binder L.I. (1986). Abnormal Phosphorylation of the Microtubule-Associated Protein Tau (Tau) in Alzheimer Cytoskeletal Pathology. Proc. Natl. Acad. Sci. USA.

[77] Frost B., Jacks R.L., Diamond M.I. (2009). Propagation of Tau Misfolding from the Outside to the Inside of a Cell. J. Biol. Chem..

[78] Nelson P.T., Alafuzoff I., Bigio E.H., Bouras C., Braak H., Cairns N.J., Castellani R.J., Crain B.J., Davies P., Tredici K.D. (2012). Correlation of Alzheimer Disease Neuropathologic Changes With Cognitive Status: A Review of the Literature. J. Neuropathol. Exp. Neurol..

[79] Ittner L.M., Ke Y.D., Delerue F., Bi M., Gladbach A., van Eersel J., Wölfing H., Chieng B.C., Christie M.J., Napier I.A. (2010). Dendritic Function of Tau Mediates Amyloid-β Toxicity in Alzheimer’s Disease Mouse Models. Cell.

[80] Small S.A., Duff K. (2008). Linking Aβ and Tau in Late-Onset Alzheimer’s Disease: A Dual Pathway Hypothesis. Neuron.

[81] Kametani F., Hasegawa M. (2018). Reconsideration of Amyloid Hypothesis and Tau Hypothesis in Alzheimer’s Disease. Front. Neurosci..

[82] Maccioni R.B., Farías G., Morales I., Navarrete L. (2010). The Revitalized Tau Hypothesis on Alzheimer’s Disease. Arch. Med. Res..

[83] Liu Y., Nguyen M., Robert A., Meunier B. (2019). Metal Ions in Alzheimer’s Disease: A Key Role or Not?. Acc. Chem. Res..

[84] Singh S.K., Balendra V., Obaid A.A., Esposto J., Tikhonova M.A., Gautam N.K., Poeggeler B. (2022). Copper-Mediated β-Amyloid Toxicity and Its Chelation Therapy in Alzheimer’s Disease. Met. Integr. Biomet. Sci..

[85] Wang L., Yin Y.-L., Liu X.-Z., Shen P., Zheng Y.-G., Lan X.-R., Lu C.-B., Wang J.-Z. (2020). Current Understanding of Metal Ions in the Pathogenesis of Alzheimer’s Disease. Transl. Neurodegener..

[86] Stelmashook E.V., Isaev N.K., Genrikhs E.E., Amelkina G.A., Khaspekov L.G., Skrebitsky V.G., Illarioshkin S.N. (2014). Role of Zinc and Copper Ions in the Pathogenetic Mechanisms of Alzheimer’s and Parkinson’s Diseases. Biochem. Mosc..

[87] Chen L.-L., Fan Y.-G., Zhao L.-X., Zhang Q., Wang Z.-Y. (2023). The Metal Ion Hypothesis of Alzheimer’s Disease and the Anti-Neuroinflammatory Effect of Metal Chelators. Bioorganic Chem..

[88] Arnsten A.F.T., Datta D., Del Tredici K., Braak H. (2021). Hypothesis: Tau Pathology Is an Initiating Factor in Sporadic Alzheimer’s Disease. Alzheimers Dement..

[89] De Strooper B., Karran E. (2016). The Cellular Phase of Alzheimer’s Disease. Cell.

[90] Liu P.-P., Xie Y., Meng X.-Y., Kang J.-S. (2019). History and Progress of Hypotheses and Clinical Trials for Alzheimer’s Disease. Signal Transduct. Target. Ther..

[91] Francis P.T., Palmer A.M., Snape M., Wilcock G.K. (1999). The Cholinergic Hypothesis of Alzheimer’s Disease: A Review of Progress. J. Neurol. Neurosurg. Psychiatry.

[92] Swerdlow R.H., Burns J.M., Khan S.M. (2014). The Alzheimer’s Disease Mitochondrial Cascade Hypothesis: Progress and Perspectives. Biochim. Biophys. Acta BBA Mol. Basis Dis..

[93] Heppner F.L., Ransohoff R.M., Becher B. (2015). Immune Attack: The Role of Inflammation in Alzheimer Disease. Nat. Rev. Neurosci..

[94] Di Marco L.Y., Venneri A., Farkas E., Evans P.C., Marzo A., Frangi A.F. (2015). Vascular Dysfunction in the Pathogenesis of Alzheimer’s Disease—A Review of Endothelium-Mediated Mechanisms and Ensuing Vicious Circles. Neurobiol. Dis..

[95] Soto-Rojas L.O., Pacheco-Herrero M., Martínez-Gómez P.A., Campa-Córdoba B.B., Apátiga-Pérez R., Villegas-Rojas M.M., Harrington C.R., de la Cruz F., Garcés-Ramírez L., Luna-Muñoz J. (2021). The Neurovascular Unit Dysfunction in Alzheimer’s Disease. Int. J. Mol. Sci..

[96] Elbert D.L., Patterson B.W., Lucey B.P., Benzinger T.L.S., Bateman R.J. (2022). Importance of CSF-Based Aβ Clearance with Age in Humans Increases with Declining Efficacy of Blood-Brain Barrier/Proteolytic Pathways. Commun. Biol..

[97] Bu X.-L., Xiang Y., Jin W.-S., Wang J., Shen L.-L., Huang Z.-L., Zhang K., Liu Y.-H., Zeng F., Liu J.-H. (2018). Blood-Derived Amyloid-β Protein Induces Alzheimer’s Disease Pathologies. Mol. Psychiatry.

[98] Fuller J.T., Cronin-Golomb A., Gatchel J.R., Norton D.J., Guzmán-Vélez E., Jacobs H.I.L., Hanseeuw B., Pardilla-Delgado E., Artola A., Baena A. (2019). Biological and Cognitive Markers of Presenilin1 E280a Autosomal Dominant Alzheimer’s Disease: A Comprehensive Review of the Colombian Kindred. J. Prev. Alzheimers Dis..

[99] Ávila-Villanueva M., Marcos Dolado A., Gómez-Ramírez J., Fernández-Blázquez M. (2022). Brain Structural and Functional Changes in Cognitive Impairment Due to Alzheimer’s Disease. Front. Psychol..

[100] Porsteinsson A.P., Isaacson R.S., Knox S., Sabbagh M.N., Rubino I. (2021). Diagnosis of Early Alzheimer’s Disease: Clinical Practice in 2021. J. Prev. Alzheimers Dis..

[101] van der Schaar J., Visser L.N.C., Bouwman F.H., Ket J.C.F., Scheltens P., Bredenoord A.L., van der Flier W.M. (2022). Considerations Regarding a Diagnosis of Alzheimer’s Disease before Dementia: A Systematic Review. Alzheimers Res. Ther..

[102] Davenport F., Gallacher J., Kourtzi Z., Koychev I., Matthews P.M., Oxtoby N.P., Parkes L.M., Priesemann V., Rowe J.B., Smye S.W. (2023). Neurodegenerative Disease of the Brain: A Survey of Interdisciplinary Approaches. J. R. Soc. Interface.

[103] Zhou Y., Song Z., Han X., Li H., Tang X. (2021). Prediction of Alzheimer’s Disease Progression Based on Magnetic Resonance Imaging. ACS Chem. Neurosci..

[104] Turner R.S., Stubbs T., Davies D.A., Albensi B.C. (2020). Potential New Approaches for Diagnosis of Alzheimer’s Disease and Related Dementias. Front. Neurol..

[105] Ossenkoppele R., van der Kant R., Hansson O. (2022). Tau Biomarkers in Alzheimer’s Disease: Towards Implementation in Clinical Practice and Trials. Lancet Neurol..

[106] Gauthier S., Rosa-Neto P., Morais J.A., Webster C. World Alzheimer Report 2021: Journey through the Diagnosis of Dementia 2021. https://www.alzint.org/u/World-Alzheimer-Report-2021.pdf.

[107] Nemy M., Cedres N., Grothe M.J., Muehlboeck J.-S., Lindberg O., Nedelska Z., Stepankova O., Vyslouzilova L., Eriksdotter M., Barroso J. (2020). Cholinergic White Matter Pathways Make a Stronger Contribution to Attention and Memory in Normal Aging than Cerebrovascular Health and Nucleus Basalis of Meynert. NeuroImage.

[108] Cedres N., Ferreira D., Nemy M., Machado A., Pereira J.B., Shams S., Wahlund L.-O., Zettergren A., Stepankova O., Vyslouzilova L. (2022). Association of Cerebrovascular and Alzheimer Disease Biomarkers With Cholinergic White Matter Degeneration in Cognitively Unimpaired Individuals. Neurology.

[109] Ossenkoppele R., Pichet Binette A., Groot C., Smith R., Strandberg O., Palmqvist S., Stomrud E., Tideman P., Ohlsson T., Jögi J. (2022). Amyloid and Tau PET-Positive Cognitively Unimpaired Individuals Are at High Risk for Future Cognitive Decline. Nat. Med..

[110] Wang C., Xu T., Yu W., Li T., Han H., Zhang M., Tao M. (2022). Early Diagnosis of Alzheimer’s Disease and Mild Cognitive Impairment Based on Electroencephalography: From the Perspective of Event Related Potentials and Deep Learning. Int. J. Psychophysiol..

[111] Blennow K., Shaw L.M., Stomrud E., Mattsson N., Toledo J.B., Buck K., Wahl S., Eichenlaub U., Lifke V., Simon M. (2019). Predicting Clinical Decline and Conversion to Alzheimer’s Disease or Dementia Using Novel Elecsys Aβ(1–42), PTau and TTau CSF Immunoassays. Sci. Rep..

[112] Veitch D.P., Weiner M.W., Aisen P.S., Beckett L.A., DeCarli C., Green R.C., Harvey D., Jack C.R., Jagust W., Landau S.M. (2022). Using the Alzheimer’s Disease Neuroimaging Initiative to Improve Early Detection, Diagnosis, and Treatment of Alzheimer’s Disease. Alzheimers Dement..

[113] DeMarco M.L., Nguyen Q., Fok A., Hsiung G.R., Gugten J.G. (2020). An Automated Clinical Mass Spectrometric Method for Identification and Quantification of Variant and Wild-type Amyloid-β 1-40 and 1-42 Peptides in CSF. Alzheimers Dement. Diagn. Assess. Dis. Monit..

[114] Esquivel R.N., Benina N., Hawkins D.M., De Simone F., Le Bastard N., Vandijck M., Gannon S., Latham J., Radwan R.R., Dickson D. (2021). Clinical Validation of the Lumipulse *G* Β-amyloid Ratio (1-42/1-40) in a Subset of ADNI CSF Samples. Alzheimers Dement..

[115] Shea D., Colasurdo E., Smith A., Paschall C., Jayadev S., Keene C.D., Galasko D., Ko A., Li G., Peskind E. (2022). SOBA: Development and Testing of a Soluble Oligomer Binding Assay for Detection of Amyloidogenic Toxic Oligomers. Proc. Natl. Acad. Sci. USA.

[116] Habashi M., Vutla S., Tripathi K., Senapati S., Chauhan P.S., Haviv-Chesner A., Richman M., Mohand S.-A., Dumulon-Perreault V., Mulamreddy R. (2022). Early Diagnosis and Treatment of Alzheimer’s Disease by Targeting Toxic Soluble Aβ Oligomers. Proc. Natl. Acad. Sci. USA.

[117] Ashton N.J., Janelidze S., Mattsson-Carlgren N., Binette A.P., Strandberg O., Brum W.S., Karikari T.K., González-Ortiz F., Di Molfetta G., Meda F.J. (2022). Differential Roles of Aβ42/40, p-Tau231 and p-Tau217 for Alzheimer’s Trial Selection and Disease Monitoring. Nat. Med..

[118] Tatulian S.A. (2022). Challenges and Hopes for Alzheimer’s Disease. Drug Discov. Today.

[119] Sang Z., Wang K., Dong J., Tang L. (2022). Alzheimer’s Disease: Updated Multi-Targets Therapeutics Are in Clinical and in Progress. Eur. J. Med. Chem..

[120] Gallego Villarejo L., Bachmann L., Marks D., Brachthäuser M., Geidies A., Müller T. (2022). Role of Intracellular Amyloid β as Pathway Modulator, Biomarker, and Therapy Target. Int. J. Mol. Sci..

[121] Jeremic D., Jiménez-Díaz L., Navarro-López J.D. (2021). Past, Present and Future of Therapeutic Strategies against Amyloid-β Peptides in Alzheimer’s Disease: A Systematic Review. Ageing Res. Rev..

[122] Uddin S.M., Kabir T.M., Rahman S.M., Behl T., Jeandet P., Ashraf G.M., Najda A., Bin-Jumah M.N., El-Seedi H.R., Abdel-Daim M.M. (2020). Revisiting the Amyloid Cascade Hypothesis: From Anti-Aβ Therapeutics to Auspicious New Ways for Alzheimer’s Disease. Int. J. Mol. Sci..

[123] Ashrafian H., Zadeh E.H., Khan R.H. (2021). Review on Alzheimer’s Disease: Inhibition of Amyloid Beta and Tau Tangle Formation. Int. J. Biol. Macromol..

[124] Salahuddin P., Khan R.H., Furkan M., Uversky V.N., Islam Z., Fatima M.T. (2021). Mechanisms of Amyloid Proteins Aggregation and Their Inhibition by Antibodies, Small Molecule Inhibitors, Nano-Particles and Nano-Bodies. Int. J. Biol. Macromol..

[125] Giorgetti S., Greco C., Tortora P., Aprile F. (2018). Targeting Amyloid Aggregation: An Overview of Strategies and Mechanisms. Int. J. Mol. Sci..

[126] Penke B., Bogár F., Crul T., Sántha M., Tóth E.M., Vigh L. (2018). Heat Shock Proteins and Autophagy Pathways in Neuroprotection: From Molecular Bases to Pharmacological Interventions. Int. J. Mol. Sci..

[127] Yang L., Wang W., Chen J., Wang N., Zheng G. (2018). A Comparative Study of Resveratrol and Resveratrol-Functional Selenium Nanoparticles: Inhibiting Amyloid β Aggregation and Reactive Oxygen Species Formation Properties. J. Biomed. Mater. Res. A.

[128] Santos M.A., Chand K., Chaves S. (2016). Recent Progress in Multifunctional Metal Chelators as Potential Drugs for Alzheimer’s Disease. Coord. Chem. Rev..

[129] Wang Y., Yang Y., Hong K.H., Ning Y., Yu P., Ren J., Ji M., Cai J. (2019). Design, Synthesis and Evaluation of a Novel Metal Chelator as Multifunctional Agents for the Treatment of Alzheimer’s Disease. Bioorganic Chem..

[130] Lin G., Zhu F., Kanaan N.M., Asano R., Shirafuji N., Sasaki H., Yamaguchi T., Enomoto S., Endo Y., Ueno A. (2021). Clioquinol Decreases Levels of Phosphorylated, Truncated, and Oligomerized Tau Protein. Int. J. Mol. Sci..

[131] Wang J., Wang K., Zhu Z., He Y., Zhang C., Guo Z., Wang X. (2019). Inhibition of Metal-Induced Amyloid β-Peptide Aggregation by a Blood–Brain Barrier Permeable Silica–Cyclen Nanochelator. RSC Adv..

[132] Taş K., Volta B.D., Lindner C., El Bounkari O., Hille K., Tian Y., Puig-Bosch X., Ballmann M., Hornung S., Ortner M. (2022). Designed Peptides as Nanomolar Cross-Amyloid Inhibitors Acting via Supramolecular Nanofiber Co-Assembly. Nat. Commun..

[133] Wagner J., Degenhardt K., Veit M., Louros N., Konstantoulea K., Skodras A., Wild K., Liu P., Obermüller U., Bansal V. (2022). Medin Co-Aggregates with Vascular Amyloid-β in Alzheimer’s Disease. Nature.

[134] Ganne A., Balasubramaniam M., Mainali N., Atluri P., Shmookler Reis R.J., Ayyadevara S. (2022). Physiological Consequences of Targeting 14-3-3 and Its Interacting Partners in Neurodegenerative Diseases. Int. J. Mol. Sci..

[135] Wilcock G.K., Gauthier S., Frisoni G.B., Jia J., Hardlund J.H., Moebius H.J., Bentham P., Kook K.A., Schelter B.O., Wischik D.J. (2018). Potential of Low Dose Leuco-Methylthioninium Bis(Hydromethanesulphonate) (LMTM) Monotherapy for Treatment of Mild Alzheimer’s Disease: Cohort Analysis as Modified Primary Outcome in a Phase III Clinical Trial. J. Alzheimers Dis..

[136] Asadzadeh J., Ruchti E., Jiao W., Limoni G., MacLachlan C., Small S.A., Knott G., Santa-Maria I., McCabe B.D. (2022). Retromer Deficiency in Tauopathy Models Enhances the Truncation and Toxicity of Tau. Nat. Commun..

[137] Curtis M.E., Smith T., Yu D., Praticò D. (2022). Association of Retromer Deficiency and Tau Pathology in Down Syndrome. Ann. Neurol..

[138] Martinez P., Patel H., You Y., Jury N., Perkins A., Lee-Gosselin A., Taylor X., You Y., Viana Di Prisco G., Huang X. (2022). Bassoon Contributes to Tau-Seed Propagation and Neurotoxicity. Nat. Neurosci..

[139] McLean C.A., Cherny R.A., Fraser F.W., Fuller S.J., Smith M.J., Beyreuther K., Bush A.I., Masters C.L. (1999). Soluble Pool of Abeta Amyloid as a Determinant of Severity of Neurodegeneration in Alzheimer’s Disease. Ann. Neurol..

[140] Holscher C., Gengler S., Gault V.A., Harriott P., Mallot H.A. (2007). Soluble Beta-Amyloid[25–35] Reversibly Impairs Hippocampal Synaptic Plasticity and Spatial Learning. Eur. J. Pharmacol..

[141] Bard F., Cannon C., Barbour R., Burke R.-L., Games D., Grajeda H., Guido T., Hu K., Huang J., Johnson-Wood K. (2000). Peripherally Administered Antibodies against Amyloid β-Peptide Enter the Central Nervous System and Reduce Pathology in a Mouse Model of Alzheimer Disease. Nat. Med..

[142] Song C., Shi J., Zhang P., Zhang Y., Xu J., Zhao L., Zhang R., Wang H., Chen H. (2022). Immunotherapy for Alzheimer’s Disease: Targeting β-Amyloid and Beyond. Transl. Neurodegener..

[143] Karran E., De Strooper B. (2022). The Amyloid Hypothesis in Alzheimer Disease: New Insights from New Therapeutics. Nat. Rev. Drug Discov..

[144] van Dyck C.H., Swanson C.J., Aisen P., Bateman R.J., Chen C., Gee M., Kanekiyo M., Li D., Reyderman L., Cohen S. (2023). Lecanemab in Early Alzheimer’s Disease. N. Engl. J. Med..

[145] Wang D., Chen F., Han Z., Yin Z., Ge X., Lei P. (2021). Relationship Between Amyloid-β Deposition and Blood–Brain Barrier Dysfunction in Alzheimer’s Disease. Front. Cell Neurosci..

[146] Zhang Y.-L., Wang J., Zhang Z.-N., Su Q., Guo J.-H. (2022). The Relationship between Amyloid-Beta and Brain Capillary Endothelial Cells in Alzheimer’s Disease. Neural Regen. Res..

[147] Sharma S., Behl T., Kumar A., Sehgal A., Singh S., Sharma N., Bhatia S., Al-Harrasi A., Bungau S. (2021). Targeting Endothelin in Alzheimer’s Disease: A Promising Therapeutic Approach. BioMed Res. Int..

[148] Iliff J.J., Wang M., Liao Y., Plogg B.A., Peng W., Gundersen G.A., Benveniste H., Vates G.E., Deane R., Goldman S.A. (2012). A Paravascular Pathway Facilitates CSF Flow Through the Brain Parenchyma and the Clearance of Interstitial Solutes, Including Amyloid β. Sci. Transl. Med..

[149] Verheggen I.C.M., Van Boxtel M.P.J., Verhey F.R.J., Jansen J.F.A., Backes W.H. (2018). Interaction between Blood-Brain Barrier and Glymphatic System in Solute Clearance. Neurosci. Biobehav. Rev..

[150] Mogensen F.L.-H., Delle C., Nedergaard M. (2021). The Glymphatic System (En)during Inflammation. Int. J. Mol. Sci..

[151] Riba M., del Valle J., Molina-Porcel L., Pelegrí C., Vilaplana J. (2022). Wasteosomes (Corpora Amylacea) as a Hallmark of Chronic Glymphatic Insufficiency. Proc. Natl. Acad. Sci. USA.

[152] Cunningham C., Hennessy E. (2015). Co-Morbidity and Systemic Inflammation as Drivers of Cognitive Decline: New Experimental Models Adopting a Broader Paradigm in Dementia Research. Alzheimers Res. Ther..

[153] Turner M.D., Nedjai B., Hurst T., Pennington D.J. (2014). Cytokines and Chemokines: At the Crossroads of Cell Signalling and Inflammatory Disease. Biochim. Biophys. Acta BBA Mol. Cell Res..

[154] Rauf A., Badoni H., Abu-Izneid T., Olatunde A., Rahman M.M., Painuli S., Semwal P., Wilairatana P., Mubarak M.S. (2022). Neuroinflammatory Markers: Key Indicators in the Pathology of Neurodegenerative Diseases. Molecules.

[155] Chen L., Deng H., Cui H., Fang J., Zuo Z., Deng J., Li Y., Wang X., Zhao L. (2018). Inflammatory Responses and Inflammation-Associated Diseases in Organs. Oncotarget.

[156] Penke B., Fulop L., Szucs M., Frecska E. (2017). The Role of Sigma-1 Receptor, an Intracellular Chaperone in Neurodegenerative Diseases. Curr. Neuropharmacol..

[157] Ahmad M.A., Kareem O., Khushtar M., Akbar M., Haque M.R., Iqubal A., Haider M.F., Pottoo F.H., Abdulla F.S., Al-Haidar M.B. (2022). Neuroinflammation: A Potential Risk for Dementia. Int. J. Mol. Sci..

[158] de Oliveira J., Kucharska E., Garcez M.L., Rodrigues M.S., Quevedo J., Moreno-Gonzalez I., Budni J. (2021). Inflammatory Cascade in Alzheimer’s Disease Pathogenesis: A Review of Experimental Findings. Cells.

[159] Szabo A., O’Connell K.S., Ueland T., Sheikh M.A., Agartz I., Andreou D., Aukrust P., Boye B., Bøen E., Drange O.K. (2022). Increased Circulating IL-18 Levels in Severe Mental Disorders Indicate Systemic Inflammasome Activation. Brain. Behav. Immun..

[160] Cheng X., Wei Y., Qian Z., Han L. Autophagy Balances Neuroinflammation in Alzheimer’s Disease. Cell Mol. Neurobiol..

[161] Panda C., Mahapatra R.K. (2023). Bi-Directional Relationship Between Autophagy and Inflammasomes in Neurodegenerative Disorders. Cell Mol. Neurobiol..

[162] Derecki N.C., Katzmarski N., Kipnis J., Meyer-Luehmann M. (2014). Microglia as a Critical Player in Both Developmental and Late-Life CNS Pathologies. Acta Neuropathol..

[163] Fekete R., Cserép C., Lénárt N., Tóth K., Orsolits B., Martinecz B., Méhes E., Szabó B., Németh V., Gönci B. (2018). Microglia Control the Spread of Neurotropic Virus Infection via P2Y12 Signalling and Recruit Monocytes through P2Y12-Independent Mechanisms. Acta Neuropathol..

[164] Herdy J.R., Traxler L., Agarwal R.K., Karbacher L., Schlachetzki J.C.M., Boehnke L., Zangwill D., Galasko D., Glass C.K., Mertens J. (2022). Increased Post-Mitotic Senescence in Aged Human Neurons Is a Pathological Feature of Alzheimer’s Disease. Cell Stem Cell.

[165] McNamara N.B., Munro D.A.D., Bestard-Cuche N., Uyeda A., Bogie J.F.J., Hoffmann A., Holloway R.K., Molina-Gonzalez I., Askew K.E., Mitchell S. (2023). Microglia Regulate Central Nervous System Myelin Growth and Integrity. Nature.

[166] Cserép C., Pósfai B., Lénárt N., Fekete R., László Z.I., Lele Z., Orsolits B., Molnár G., Heindl S., Schwarcz A.D. (2020). Microglia Monitor and Protect Neuronal Function through Specialized Somatic Purinergic Junctions. Science.

[167] Spangenberg E., Severson P.L., Hohsfield L.A., Crapser J., Zhang J., Burton E.A., Zhang Y., Spevak W., Lin J., Phan N.Y. (2019). Sustained Microglial Depletion with CSF1R Inhibitor Impairs Parenchymal Plaque Development in an Alzheimer’s Disease Model. Nat. Commun..

[168] Sierksma A., Lu A., Mancuso R., Fattorelli N., Thrupp N., Salta E., Zoco J., Blum D., Buée L., De Strooper B. (2020). Novel Alzheimer Risk Genes Determine the Microglia Response to Amyloid-β but Not to TAU Pathology. EMBO Mol. Med..

[169] Sala Frigerio C., Wolfs L., Fattorelli N., Thrupp N., Voytyuk I., Schmidt I., Mancuso R., Chen W.-T., Woodbury M.E., Srivastava G. (2019). The Major Risk Factors for Alzheimer’s Disease: Age, Sex, and Genes Modulate the Microglia Response to Aβ Plaques. Cell Rep..

[170] Andreasson K.I., Bachstetter A.D., Colonna M., Ginhoux F., Holmes C., Lamb B., Landreth G., Lee D.C., Low D., Lynch M.A. (2016). Targeting Innate Immunity for Neurodegenerative Disorders of the Central Nervous System. J. Neurochem..

[171] Chen Y., Colonna M. (2021). Microglia in Alzheimer’s Disease at Single-Cell Level. Are There Common Patterns in Humans and Mice?. J. Exp. Med..

[172] Al-Ghraiybah N.F., Wang J., Alkhalifa A.E., Roberts A.B., Raj R., Yang E., Kaddoumi A. (2022). Glial Cell-Mediated Neuroinflammation in Alzheimer’s Disease. Int. J. Mol. Sci..

[173] Dhapola R., Hota S.S., Sarma P., Bhattacharyya A., Medhi B., Reddy D.H. (2021). Recent Advances in Molecular Pathways and Therapeutic Implications Targeting Neuroinflammation for Alzheimer’s Disease. Inflammopharmacology.

[174] Zelová H., Hošek J. (2013). TNF-α Signalling and Inflammation: Interactions between Old Acquaintances. Inflamm. Res..

[175] Ennerfelt H., Frost E.L., Shapiro D.A., Holliday C., Zengeler K.E., Voithofer G., Bolte A.C., Lammert C.R., Kulas J.A., Ulland T.K. (2022). SYK Coordinates Neuroprotective Microglial Responses in Neurodegenerative Disease. Cell.

[176] Puntambekar S.S., Moutinho M., Lin P.B.-C., Jadhav V., Tumbleson-Brink D., Balaji A., Benito M.A., Xu G., Oblak A., Lasagna-Reeves C.A. (2022). CX3CR1 Deficiency Aggravates Amyloid Driven Neuronal Pathology and Cognitive Decline in Alzheimer’s Disease. Mol. Neurodegener..

[177] Pereira J.B., Janelidze S., Strandberg O., Whelan C.D., Zetterberg H., Blennow K., Palmqvist S., Stomrud E., Mattsson-Carlgren N., Hansson O. (2022). Microglial Activation Protects against Accumulation of Tau Aggregates in Nondemented Individuals with Underlying Alzheimer’s Disease Pathology. Nat. Aging.

[178] Claes C., England W.E., Danhash E.P., Kiani Shabestari S., Jairaman A., Chadarevian J.P., Hasselmann J., Tsai A.P., Coburn M.A., Sanchez J. (2022). The P522R Protective Variant of PLCG2 Promotes the Expression of Antigen Presentation Genes by Human Microglia in an Alzheimer’s Disease Mouse Model. Alzheimers Dement..

[179] Castranio E.L., Hasel P., Haure-Mirande J., Ramirez Jimenez A.V., Hamilton B.W., Kim R.D., Glabe C.G., Wang M., Zhang B., Gandy S. Microglial *INPP5D* Limits Plaque Formation and Glial Reactivity in the PSAPP Mouse Model of Alzheimer’s Disease. Alzheimers Dement..

[180] Birch A.M. (2014). The Contribution of Astrocytes to Alzheimer’s Disease. Biochem. Soc. Trans..

[181] Zhang Y., Bailey J.T., Xu E., Singh K., Lavaert M., Link V.M., D’Souza S., Hafiz A., Cao J., Cao G. (2022). Mucosal-Associated Invariant T Cells Restrict Reactive Oxidative Damage and Preserve Meningeal Barrier Integrity and Cognitive Function. Nat. Immunol..

[182] Sasaguri H., Hashimoto S., Watamura N., Sato K., Takamura R., Nagata K., Tsubuki S., Ohshima T., Yoshiki A., Sato K. (2022). Recent Advances in the Modeling of Alzheimer’s Disease. Front. Neurosci..

